# Relationships between COVID-19 healthcare outcomes and county characteristics in the U.S. for Delta (B.1.617.2) and Omicron (B.1.1.529 and BA.1.1) variants

**DOI:** 10.3389/fpubh.2023.1252668

**Published:** 2023-11-17

**Authors:** Alexander A. Bruckhaus, Yujia Zhang, Sana Salehi, Aidin Abedi, Dominique Duncan

**Affiliations:** ^1^Laboratory of Neuro Imaging, USC Stevens Neuroimaging and Informatics Institute, Keck School of Medicine of USC, University of Southern California, Los Angeles, CA, United States; ^2^USC Neurorestoration Center, Keck School of Medicine, University of Southern California, Los Angeles, CA, United States; ^3^Department of Neurological Surgery, Keck School of Medicine, University of Southern California, Los Angeles, CA, United States; ^4^Rancho Research Institute, Rancho Los Amigos National Rehabilitation Center, Downey, CA, United States

**Keywords:** COVID-19, public health, socioeconomic factors, regression analysis, COVID-19 vaccines

## Abstract

**Background:**

COVID-19 is constantly evolving, and highly populated communities consist of many different characteristics that may contribute to COVID-19 health outcomes. Therefore, we aimed to (1) quantify the relationships between county characteristics and severe and non-severe county-level health outcomes related to COVID-19. We also aimed to (2) compare these relationships across time periods where the Delta (B.1.617.2) and Omicron (B.1.1.529 and BA.1.1) variants were dominant in the U.S.

**Methods:**

We used multiple regression to measure the strength of relationships between healthcare outcomes and county characteristics in the 50 most populous U.S. counties.

**Results:**

We found many different significant predictors including the proportion of a population vaccinated, median household income, population density, and the proportion of residents aged 65+, but mainly found that socioeconomic factors and the proportion of a population vaccinated play a large role in the dynamics of the spread and severity of COVID-19 in communities with high populations.

**Discussion:**

The present study shines light on the associations between public health outcomes and county characteristics and how these relationships change throughout Delta and Omicron’s dominance. It is important to understand factors underlying COVID-19 health outcomes to prepare for future health crises.

## 1 Introduction

As new variants of the Severe Acute Respiratory Syndrome Coronavirus 2 (SARS-CoV-2) evolve and mutate, it is vital to understand how these new variants affect public health outcomes. Our understanding of COVID-19 can have significant impacts on how we approach measures to mitigate the virus’s deleterious effects on communities.

Before the Omicron variant (B.1.1.529 and BA.1.1) became the dominant strain in the United States (U.S.), the Delta variant (B.1.617.2) existed in the U.S. from late June 2021 to late December 2021 as the dominant strain (1). It has been reported to be up to over two times more contagious than preceding variants (2–5) along with increased severity (2, 4).

In what became the newest variant of concern (VOC) at the time, the first confirmed case of the Omicron variant in the U.S was discovered on 1 December 2021 (6). Data show that the Omicron variant became dominant in the U.S. only about 3 weeks after the first confirmed case, approximately on 19 December 2021 according to weekly reports (1). While Omicron’s expeditious rise to dominance points to higher transmission levels relative to preceding variants, numerous studies also highlight the variant’s highly transmissive nature (2, 7–9). Studies suggest that the Omicron variant can infect three to six times as many people as the Delta variant over the same time period (7, 8, 10). However, it is reported to cause less severe symptoms (10). In the present study, the two lineages that make up the Omicron variant are B.1.1.529 and BA.1.1, which accounted for the majority of COVID-19 cases in the U.S. from approximately 19 December 2021 to the study end date (19 March 2022).

COVID-19 has historically been dubbed as “the great equalizer” (11) as the disease purportedly transcends wealth, fame, prestige, and age. However, there exists nuance surrounding this line of reasoning that shines light on the impact of social, economic, and demographic characteristics in communities.

The social, economic, and demographic characteristics of communities are largely cited to affect macro-level health outcomes and contribute to several indices that aim to draw relationships between regional characteristics and health outcomes (12–16). Although the variables included in the present study were drawn from more than one existing framework, careful consideration of each variable and its empirical implications were taken.

The common denominator among a vast amount of epidemiological COVID-19 studies are: socioeconomic, demographic, and environmental/behavioral factors (12–16). Therefore, we aimed to include core variables from each aforementioned facet of a community’s makeup in order to examine their relationship with the non-severe and severe outcomes in highly populated regions, over the course of the virus’s evolution into new variants. We also included the variable of the proportion of residents vaccinated to address the effect of community-wide interventional measures on health outcomes. Median household income represented the socioeconomic facet of a community’s make up as it has been shown in other studies to have one of the highest relative effects on COVID-19 case and death rates (15, 17–22). The proportion of residents aged 65 and older are included to represent the demographic facet of a community’s make up, as it has been reported that individuals aged 65 and older are at a higher risk for more severe outcomes from COVID-19 and contribute to much of COVID-19 related deaths (23). Lastly, population density, although not widely reported in vulnerability indices like the CDC vulnerability index (SVI), has been shown to have differing effects overtime (24–26) and is reported as imperative to investigate in the context of COVID-19 (14) as not all current vulnerability indices incorporate this measure. Altogether, these independent variables each represent a core aspect (socioeconomic, demographic, environmental, and interventional) of a community’s risk for non-severe and severe health outcomes. Although case rate is a primary indicator of non-severe macro-level health outcomes, we also included positivity rate and infection rate as additional indicators in order to deeper investigate non-severe macro-level health outcomes. By a similar token for severe health outcomes, deaths are also a primary indicator, but we also included hospitalizations, ICU occupancies, and ICU occupancy from COVID-19 to deeper explore severe macro-level health outcomes.

SARS-CoV-2 variants are reported to have differing levels of effects, with the Delta variant being more severe and the Omicron variant being more transmissive (6, 27). Therefore, we aimed to measure the relationships of the aforementioned independent variables with macro-level health outcomes to observe how these relationships change in different periods of variant dominance. With respect to the proportion of residents vaccinated, as the COVID-19 vaccine has been reported to wane in effectiveness from Delta to the Omicron variant (28), it becomes valuable to investigate the macro-level relationship of vaccines and community health outcomes. By the same token, investigating the relationship of the proportion of residents aged older than 65 and community health outcomes may provide insight into the distinct variant’s macro-level effects on the older adult population. In the case of population density, its effect on macro-level health outcomes has been shown to change over time (27), thus making it valuable to investigate inter-variant trends. Socioeconomic trends are also vital to monitor throughout the time course of different variant dominances as findings may implicate the necessity for assistance programs and policy-changes to support equitable health outcomes.

Given the multifactorial etiology of the different macro-level health outcomes related to COVID-19, along with its mutative behavior, we aimed to (1) investigate the relationship between county-level characteristics and county-wide COVID-19 health outcomes pertaining to transmission and severity, and (2) investigate how these trends change over the evolution of new SARS-CoV-2 variants. We used multiple regression models to investigate the relationship between the aforementioned independent variables and public health outcomes in the 50 most populated U.S. counties over different periods of SARS-CoV-2 variant dominance. Overall, this study aims to elucidate COVID-19 trends on a macro-level and their possible implications for advancing public health knowledge and informed decision making.

## 2 Materials and methods

### 2.1 Sample

The study sample consisted of the 50 most populous counties in the U.S according to the 2020 U.S. census estimates (29).

### 2.2 Variables of interest and data definitions

#### 2.2.1 Independent variables

The independent variables used in this study were: proportion of residents with 2+ doses (or one dose of Johnson & Johnson), MHI, population density, and the proportion of residents aged 65 and older. See Table 1 for expounded descriptions of variables.

**Table 1:** Definitions and sources of variables.

	Definition	Source
**Independent variables**		
Proportion of residents vaccinated (2+ doses or J & J)	The proportion of residents in each county who have received 2+ doses or a J&J dose, reported daily and averaged across time period.	COVID Act Now API
Median household income	The median household income of a respective county	USDA
Population Density	The number of people per square mile	CDC county view
Proportion of residents aged 65+	The number of residents aged 65+ divided by the population estimate of the respective county	CDC pop 65
**Dependent variables**		
**Group 1 (severe)**		
New deaths per 100,000 residents	The number of new deaths per day, divided by the 2020 population estimate of the respective county, multiplied by 100,000.	See note
Hospitalizations per 100,000 residents	The number of residents occupying a hospital bed due to a COVID-19 case (reported every 7 days), divided by the 2020 population estimate of the respective county, multiplied by 100,000	See note
Proportion of ICU beds in use	The total number of ICU beds being used divided by the ICU bed capacity of a county (reported every 7 days)	COVID Act Now API
Proportion of ICU beds used for COVID-19	The total number of ICU beds being used for COVID-19 divided by the total number of ICU beds being used in a county (reported every 7 days)	COVID Act Now API
**Group 2 (non-severe)**		
Positivity rate	The ratio of people who test positive using a 7-day rolling average	COVID Act Now API
Infection rate	The approximate number of infections arising from a typical case	COVID Act Now API
Case density	The number of cases per 100,000 population calculated using a 7-day rolling average	COVID Act Now API

Definitions and sources of independent and dependent variables used in the study. Group 1 variables are variables taken from COVID Act Now and then modified using the 2020 census population estimates (29). All metrics used in the study are averaged during the respective time frame (Delta 1, which is from 20 June 2021 to 18 September 2021; Delta 2, which is from 19 September 2021 to 18 December 2021; and Omicron, which spans from 19 December 2021 to 19 March 2022). Median household income is retrieved from USDA (30). Group 2 variables are variables that are taken directly from the COVID Act Now data using the COVID Act Now API (30).

#### 2.2.2 Dependent variables

Among non-severe public health outcomes, we investigated the average positivity rate, average infection rate, and the average number of cases per 100,000 persons in a county (using an average of a seven-day rolling average; case density) within each time frame. Among severe outcomes, we investigated average deaths and hospitalizations per 100,000 persons, average ICU occupancy (reported every 7 days), and average proportion of ICU admissions related to COVID-19 (reported every 7 days) within each time frame. See Table 1 for expounded descriptions of variables.

### 2.3 Data acquisition

Data pertaining to case density, positivity rate, infection rate, and proportion of ICU beds in use and the proportion of ICU beds that are being used for COVID-19 of a county were obtained from COVID Act Now (31) and data pertaining to new deaths and hospitalizations per 100,000 residents were gathered from COVID Act Now (31), then modified using population data from the 2020 census (29). The vaccination data for New York County were unavailable from COVID Act Now and were therefore retrieved from CDC (32). Vaccination data from Texas were obtained from COVID Act Now until 22 October 2021, and then retrieved from the CDC thereafter because of unavailability.

### 2.4 Data curation

New deaths and hospitalizations per 100,000 residents were calculated by dividing the metric of interest (e.g., new deaths per day) by the 2020 population estimate of the respective county (29) and then multiplying this value by 100,000. All variables that were reported periodically across each period were averaged.

### 2.5 Time periods

The present study was split into three time periods, each consisting of 91 days, in which either the Delta or Omicron variant was dominant. The first two time periods (Delta 1 and Delta 2) both represent when Delta was dominant in the U.S. but was split into two time periods to allow for a more uniform comparison to Omicron’s period of dominance.

#### 2.5.1 Delta variant dominance

Delta 1 spanned from 20 June 2021 to 18 September 2021. Delta 2 spanned from 19 September 2021 to 18 December 2021. According to the COVID Data Tracker by CDC (1), the Delta variant accounted for ~37.5% of all cases in the U.S. on 19 June 2021 and 55% by 26 June 2021, therefore making it the dominant strain in the U.S. as early as 20 June 2021. This time frame ends on 19 September 2021, as this date was determined to divide Delta’s dominance into two separate time frames of nearly congruent data. That is, both the first and second time frames had a similar average proportion of Delta cases, which was ~90.6% and ~95.7%, respectively. The second time frame (Delta 2) ends on 18 December 2021. 19 December 2021, is the first possible day where Omicron could become the dominant strain in the U.S. as the Omicron variant accounted for ~37.9% of all U.S. cases on 18 December 2021, and ~77% by 25 December 2021 (1).

#### 2.5.2 Omicron variant dominance

Omicron dominance spanned from 19 December 2021 to 19 March 2022 (study end date). When the study ended, B.1.1.529 and BA.1.1 were the most dominant, which share similar characteristics in terms of transmissibility and severity (32). The BA.2 and BA.2.12.1 lineages have been identified to be slightly more contagious than previous Omicron lineages but became dominant after the study end date (33).

### 2.6 Data analysis

Data across each period were averaged, with the numerator being the sum of all daily or weekly recorded variables and the denominator being the total number of time points the data were recorded for each time period.

For each time frame, we used the ggpubr and the stats library in RStudio (version 4.0.4) (34) to perform multiple regression analysis between each dependent variable and all four independent variables. During Delta 1, Florida changed their reporting standards and thus the data was not compatible for analysis (32), so mean new deaths per 100,000 residents analysis was performed using 43 counties. The value of *p* of the association between the independent variable and the dependent variable, coefficient estimates, 95% confidence intervals for coefficient estimates, R^2^, and *p*-values of each of the multiple regression models were recorded. *p*-values smaller than 0.05 were considered statistically significant.

## 3 Results

**Figure 1:**
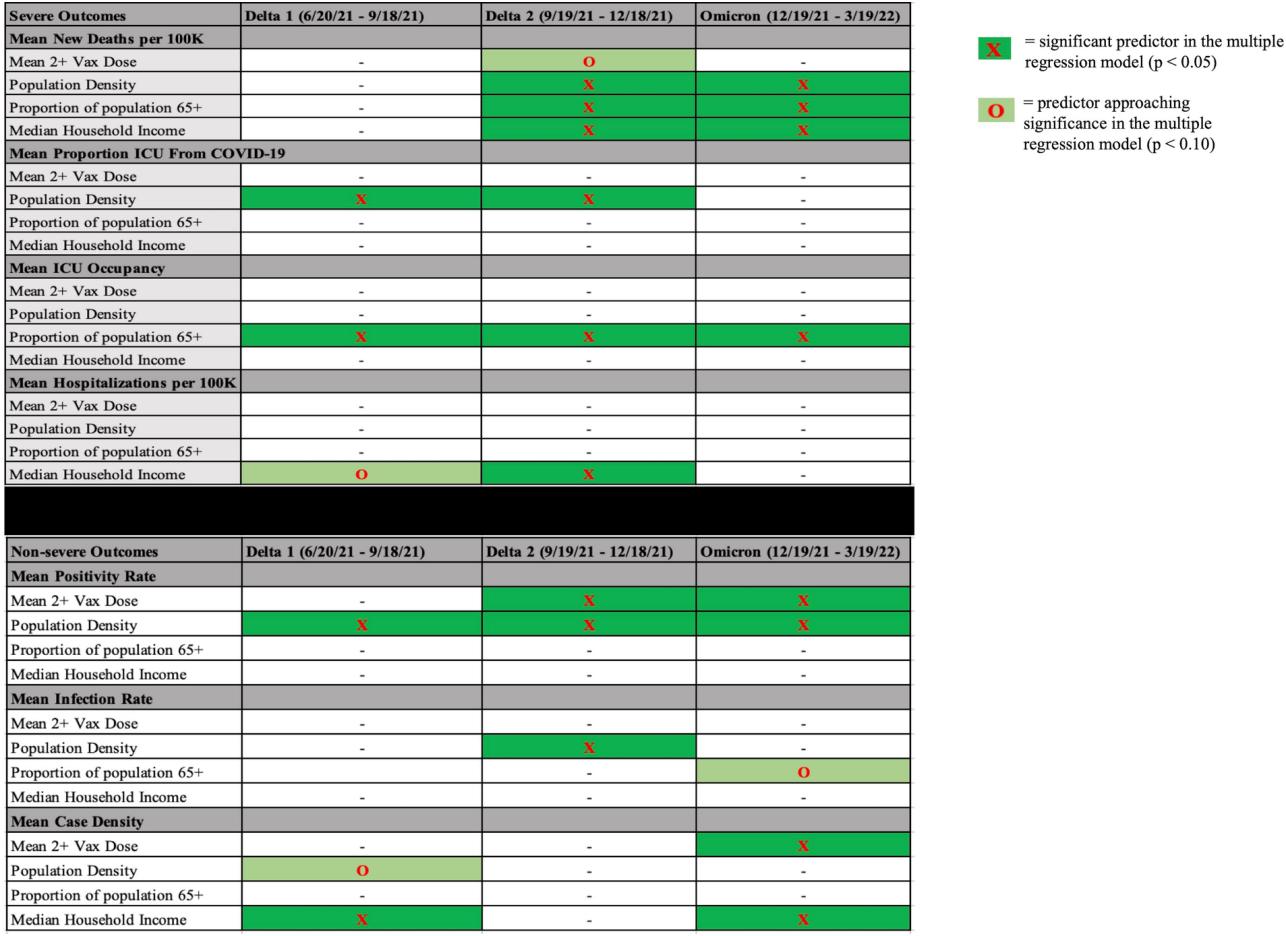
Significant predictors (or marginally significant) during each time period for each dependent variable. Significant predictors (*p* < 0.05, dark green highlight) or predictors approaching significance (*p* < 0.10, light green highlight) in each time period in each multiple regression model with severe outcomes on the top and non-severe outcomes on the bottom. Mean 2+ Vax Dose = mean proportion of residents vaccinated in a county over the course of variant dominance.

See Figure 1 for a display of findings of significant predictors across time periods and Supplementary Table A.1 for findings regarding simple regression results.

### 3.1 Delta 1 (20 June 2021 to 18 September 2021)

#### 3.1.1 Non-severe outcomes

In the mean case density model, MHI was a significant predictor (coefficient estimate (CE) = −0.000292, 95% Confidence Interval (CI) [−0.000567, −0.0000170]) while population density trended toward significance (CE = −0.000315, 95% CI [−0.000662, 0.0000310]). In the mean positivity rate model, population density was a significant predictor (CE = −0.00000109, 95% CI [−0.00000194, −0.000000226]). The mean infection rate model yielded no significant predictors.

#### 3.1.2 Severe outcomes

In the mean ICU occupancy model, the proportion of residents aged 65+ was significant (CE = −0.0128, 95% CI [−0.0235, −0.00209]). In the mean hospitalizations from COVID-19 per 100,000 residents model, MHI trended toward significance (CE = −0.000225, 95% CI [−0.000483, 0.0000334]), while population density was significant (CE = −0.0000032, 95% CI [−0.00000551, −0.0000000890]) in the mean proportion of ICU admissions due to COVID-19 model. The mean new deaths per 100,000 residents model resulted in no significant predictors. See Table 2 for all multiple regression results during Delta 1.

**Table 2:** Multiple regression results from Delta 1.

Delta 1	Mean case density	Mean new deaths per 100 k	Mean positivity rate	Mean infection rate	Mean ICU occupancy	Mean hospitalizations per 100 k	Mean proportion of ICU from COVID-19
Vax—value of *p* of predictor	0.949	0.372	0.124	0.212	0.620	0.444	0.951
Vax—coefficient estimate	2.074	−0.195	−0.128	−0.080	−0.126	−23.49	0.013
Vax—95% CI	[−63.16, 67.31]	[−0.63, 0.24]	[−0.29, 0.038]	[−0.21, 0.048]	[−0.63, 0.38]	[−84.79, 37.81]	[−0.42, 0.45]
MHI—value of *p* of predictor	0.038	0.100	0.168	0.100	0.231	0.086	0.127
MHI—coefficient estimate	−0.00029	−0.000002	−0.00000047	0.00000045	−0.000001	−0.00023	−0.000001
MHI—95% CI	[−0.00057, −0.000017]	[−0.0000034, 0.00000031]	[−0.0000012, 0.00000021]	[−0.000000089, 0.00000098]	[−0.0000034, 0.00000085]	[−0.00048, 0.000033]	[−0.0000033, 0.00000042]
PD—value of *p* of predictor	0.073	0.174	0.015	0.709	0.440	0.167	0.008
PD—coefficient estimate	−0.00032	−0.000001	−0.000001	0.00000012	−0.000001	−0.00023	−0.000003
PD—95% CI	[−0.00066, 0.000031]	[−0.0000037, 0.00000068]	[−0.0000019, −0.00000023]	[−0.00000054, 0.00000079]	[−0.0000037, 0.0000017]	[−0.00055, 0.000098]	[−0.0000055, −0.000000089]
65+—value of *p* of predictor	0.627	0.160	0.755	0.921	0.020	0.667	0.526
65+—Coefficient Estimate	0.335	−0.0082	0.00054	−0.0013	−0.013	0.279	−0.0029
65+—95% CI	[−1.05, 1.72]	[−0.02, 0.0034]	[−0.0029, 0.0040]	[−0.0028, 0.0025]	[−0.024, −0.0021]	[−1.02, 1.58]	[−0.012, 0.0063]
Adjusted R^2^ of model	0.150	0.254	0.293	−0.018	0.163	0.171	0.156
value of *p* of model	0.023	0.002	0.001	0.536	0.017	0.014	0.020
*n*	50	43	49	47	50	50	50

Vax, mean proportion of residents with 2+ doses; MHI, median household income; PD, population density; 65+, Proportion of residents aged 65 and older; *n*, sample size.

### 3.2 Delta 2 (19 September 2021 to 18 December 2021)

#### 3.2.1 Non-severe outcomes

The mean positivity rate model yielded the proportion of residents with 2+ doses (CE = −0.108, 95% CI [−0.214, −0.000781]) and population density (CE = −0.000000642, 95% CI [−0.00000127, −0.0000000174]) as a significant predictor. In the mean infection rate model, population density was a significant predictor (CE = 0.00000182, 95% CI [0.000000800, 0.00000284]). There were no significant predictors in the mean case density model.

#### 3.2.2 Severe outcomes

The mean new deaths per 100,000 model yielded three significant predictors, which were MHI (CE = −0.000004, 95% CI [−0.00000554, −0.00000246]), population density (CE = −0.0000041, 95% CI [−0.00000634, −0.00000186]), and the proportion of residents aged 65 and older (CE = 0.0123, 95% CI [0.00365, 0.0209]). The proportion of residents with 2+ doses trended toward significance (CE = −0.362, 95% CI [−0.743, 0.0187]). In the mean ICU occupancy model, the proportion of residents aged 65+ was a significant predictor (CE = −0.0142, 95% CI [−0.0226, −0.00586]). The mean hospitalizations from COVID-19 per 100,000 residents model showed that the proportion of residents with 2+ doses (CE = −33.08, 95% CI [−60.068, −6.098]) and MHI (CE = −0.000111, 95% CI [−0.000220, −0.00000136]) were significant predictors. Lastly, the mean proportion of ICU admissions due to COVID-19 showed that population density was a significant predictor (CE = −0.00000253, 95% [−0.00000409, −0.000000968]). See Table 3 for all multiple regression results during Delta 2.

**Table 3:** Multiple regression results from Delta 2.

Delta 2	Mean case density	Mean new deaths per 100 k	Mean positivity rate	Mean infection rate	Mean ICU occupancy	Mean hospitalizations per 100 k	Mean proportion of ICU from COVID-19
Vax—value of *p* of predictor	0.291	0.062	0.048	0.667	0.328	0.017	0.510
Vax—coefficient estimate	−23.570	−0.362	−0.108	0.038	−0.182	−33.080	−0.088
Vax—95% CI	[−68.04, 20.90]	[−0.74, 0.019]	[−0.21, −0.00078]	[−0.14, 0.22]	[−0.55, 0.19]	[−60.07, −6.01]	[−0.35, 0.18]
MHI—value of *p* of predictor	0.551	0.0000044	0.221	0.281	0.385	0.047	0.311
MHI—coefficient estimate	−0.000054	−0.000004	−0.00000027	0.00000039	−0.000001	−0.00011	−0.000001
MHI—95% CI	[−0.00023, 0.00013]	[−0.0000055, −0.0000025]	[−0.00000069, 0.00000017]	[−0.00000033, 0.0000011]	[−0.0000022, 0.00000084]	[−0.00022, −0.0000014]	[−0.0000016, 0.00000053]
PD—value of *p* of predictor	0.679	0.001	0.044	0.001	0.711	0.312	0.002
PD—coefficient estimate	−0.000054	−0.000004	−0.000001	0.000002	0.0000004	−0.000081	−0.000003
PD—95% CI	[−0.00032, 0.00021]	[−0.0000063, −0.0000019]	[−0.0000013, −0.000000017]	[0.00000080, 0.0000028]	[−0.0000018, 0.0000026]	[−0.00024, 0.000078]	[−0.0000041, −0.00000097]
65+—value of *p* of predictor	0.214	0.006	0.435	0.197	0.001	0.284	0.400
65+—coefficient estimate	0.630	0.012	0.00094	0.0026	−0.014	−0.329	−0.0025
65+—95% CI	[−0.38, 1.64]	[0.0037, 0.021]	[−0.0015, 0.0034]	[−0.0014, 0.0065]	[−0.023, −0.0059]	[−0.28, 0.94]	[−0.0086, 0.0035]
Adjusted R^2^ of model	0.012	0.648	0.264	0.266	0.238	0.346	0.221
value of *p* of model	0.279	0.00000000015	0.001	0.002	0.003	0.0001	0.004
*n*	50	50	49	47	50	50	50

Vax, mean proportion of residents with 2+ doses; MHI, median household income; PD, population density; 65+, Proportion of residents aged 65 and older; *n*, sample size.

### 3.3 Omicron (19 December 2021 to 19 March 2022)

#### 3.3.1 Non-severe outcomes

In the mean case density model, the proportion of residents with 2+ doses (CE = 194.8, 95% CI [100.842, 288.837]) and MHI (CE = −0.00063, 95% CI [−0.00101, −0.000240]) were significant predictors. In the mean positivity rate model, the proportion of residents with 2+ doses (CE = -0.1816, 95% CI [−0.312, −0.0512]) and population density (CE = −0.000000751, 95% CI [−0.00000150, −0.00000000180]) were significant predictors. Finally, the mean infection rate model was not significant.

#### 3.3.2 Severe outcomes

The mean new deaths per 100,000 residents model yielded three significant predictors, which were MHI (CE = −0.000003831, 95% CI [−0.00000623, −0.000001433]), population density (CE = 0.0000044, 95% CI [0.00000103, 0.00000778]), and the proportion of residents aged 65 and older (CE = 0.02548, 95% CI [0.0132, 0.0378]). The mean ICU occupancy model showed that the proportion of residents aged 65+ was a significant predictor (CE = −0.0137, 95% CI [−0.0199, −0.00746]). In the mean hospitalizations from COVID-19 per 100,000 residents model, the proportion of residents with 2+ doses (CE = −45.53, 95% CI [−82.030, −9.021]) and population density (CE = 0.000539, 95% CI [0.000328, 0.000750]) were significant predictors. Finally, the mean proportion of ICU admissions due to COVID-19 model was not significant. See Table 4 for all multiple regression results during Omicron.

**Table 4:** Multiple regression results from Omicron.

Omicron	Mean case density	Mean new deaths per 100 k	Mean positivity rate	Mean infection rate	Mean ICU occupancy	Mean hospitalizations per 100 k	Mean proportion of ICU from COVID-19
Vax—value of *p* of predictor	0.000	0.169	0.007	0.710	0.153	0.016	0.556
Vax—coefficient estimate	194.8	−0.4059	−0.1816	−0.0391	−0.21400	−45.53	−0.0704
Vax—95% CI	[100.84, 288.84]	[−0.99, 0.18]	[−0.31, −0.051]	[−0.25, 0.17]	[−0.51, 0.083]	[−82.03, −9.02]	[−0.31, 0.17]
MHI—value of *p* of predictor	0.002	0.002	0.208	0.211	0.734	0.3	0.959
MHI—coefficient estimate	−0.00063	−0.000004	−0.00000034	0.000001	−0.00000021	−0.000078	−0.000000025
MHI—95% CI	[−0.0010, −0.00024]	[−0.0000062, −0.0000014]	[−0.00000087, 0.0000002]	[−0.000000320, 0.00000141]	[−0.0000014, 0.000001]	[−0.00023, 0.000072]	[−0.000001, 0.00000096]
PD—value of *p* of predictor	0.113	0.012	0.049	0.092	0.608	0.000	0.745
PD—coefficient estimate	0.00044	0.000004	−0.000001	−0.000001	0.000000	0.000539	−0.00000022
PD—95% CI	[−0.00011, 0.00098]	[0.0000010, 0.0000078]	[−0.0000015, −0.0000000018]	[−0.0000022, 0.00000017]	[−0.0000013, 0.0000022]	[0.00033, 0.00075]	[−0.0000012, 0.0000016]
65+—value of *p* of predictor	0.404	0.00013	0.489	0.485	0.000061	0.358	0.142
65+—coefficient estimate	−0.825	0.025	−0.00094	−0.0015	−0.014	0.353	−0.0037
65+—95% CI	[−2.8, 1.15]	[0.013, 0.038]	[−0.0037, 0.0018]	[−0.0059, 0.0028]	[−0.02, −0.0075]	[−0.41, 1.12]	[−0.0088, 0.0013]
Adjusted R^2^ of model	0.353	0.532	0.43	0.066	0.343	0.444	−0.009
value of *p* of model	0.000082	0.000000078	0.0000071	0.141	0.00011	0.0000033	0.475
*n*	50	50	49	48	50	50	50

Vax, mean proportion of residents with 2+ doses; MHI, median household income; PD, population density; 65+, Proportion of residents aged 65 and older; *n*, sample size.

## 4 Discussion

To understand how community characteristics and evolving COVID-19 variants affect macro-level public health outcomes, we used multiple regression models using data from the 50 most populated counties in the U.S during the Delta and Omicron variant’s time of dominance. Our results convey relationships between county characteristics and health outcomes in the 50 most populated US counties over the three time periods. Although R^2^ values of models vary across time frames from low to moderate to high, we include all significant predictors as they may help shine light on fundamental factors affecting COVID-19 health outcomes.

### 4.1 Trends across time periods

#### 4.1.1 Non-severe outcomes

Relative to non-severe community health outcomes, our models displayed notable trends among the time frames of Delta 1, Delta 2, and Omicron.

Population density was a significant predictor of mean positivity rate in all time periods (negative coefficient estimates), corroborating that counties with higher population densities may benefit from increased infrastructure in their healthcare systems (24). Another significant predictor in the positivity rate model was the proportion of residents with 2+ COVID-19 vaccine doses (negative coefficient estimates) during the Delta 2 and Omicron time frame. If taken at face value, this points to the possibility that counties with a higher proportion of residents vaccinated achieve lower positivity rates. However, this relationship may be confounded, as one’s vaccination status could influence their likelihood to get tested. Yet, the relationship remains modestly high and may help validate the inverse relationship between vaccination and positivity rates.

Regarding the mean infection rate model, population density was a significant predictor in the Delta 2 time period (positive coefficient estimate) and trended toward significance in the Omicron time period (negative coefficient estimate). Interestingly, the positive simple regression association in Delta 2 between mean infection rate and population density (see Supplementary Table A.1 for simple regression results) seems to contradict the aforementioned theory that counties with higher population density experience better non-severe health outcomes. This finding suggests changing dynamics between the Delta 2 and Omicron time period.

Finally, in the mean case density model, MHI was a significant predictor (negative coefficient estimates) in the Delta 1 and Omicron time periods. In agreement with previous studies, this finding confirms that underlying socioeconomic factors may affect COVID-19 transmission rates (17, 18, 35). Additionally, possibly due to the more transmissive nature of the Omicron variant, the negative relationships seen between vaccination and positivity rate in Delta 2 and Omicron are not observed in the relationship of vaccination and case density in Omicron. This may be the cause of Omicron’s overwhelming initial surge, coupled with evidence of decreased vaccine effectiveness against the Omicron variant (2, 28) (yet still shown to be effective) that caused this temporary relationship.

Altogether, in the case of non-severe outcomes, on a macro-level, communities with better socio economic conditions and more vaccine access may experience better outcomes, while Omicron has indiscriminately spread faster and more resistant to the COVID-19 vaccine. Thus, the attention to community efforts of vaccination and methodical resource allocation to more socioeconomically vulnerable populations are merited.

#### 4.1.2 Severe outcomes

There were several trends among severe outcomes across Delta 1, Delta 2, and Omicron.

In the mean new deaths per 100,000 model, population density was a significant predictor during Delta 2 (negative coefficient estimate) and during Omicron (positive coefficient estimate). The initial negative relationship during Delta 2 may suggest that densely populated areas within the U.S. are better able to adapt overtime (hence the relationship during Delta 2 was significant and not Delta 1) (26). Additionally, other reports suggest that population density does not necessarily positively correlate with severe health outcomes, possibly due to advanced healthcare systems in densely populated areas in the U.S. (24, 25). However, the opposite is observed during the Omicron period. Because of Omicron’s increased levels of transmission (7, 8, 10), this observation falls in line with a CDC report which indicates that a surge in cases may lead to significant increases in hospitalizations and deaths (2), also supporting the claim that initial surges of variants affect densely populated communities more (26). MHI remains a significant predictor of new deaths across Delta 2 and Omicron (negative coefficient estimates). This may indicate that counties with a lower MHI experience more deaths possibly because of less robust healthcare systems and more public-facing occupations, while individuals residing in higher income areas may have benefitted from work-from-home practices (36) and more robust healthcare systems. Finally, the relationship between the proportion of the population aged 65+ and deaths across Delta 2 and Omicron (positive coefficient estimates) are backed up by the literature that suggests older people are at a higher risk for death from COVID-19 (23).

In the mean proportion of ICU admissions due to COVID-19 model, population density was a significant predictor across Delta 1 and Delta 2 (negative coefficient estimates), which supports the theory that counties with denser populations have greater access to resources that mitigate transmission and severity. However, it should be acknowledged that the total number of ICU beds available in a county (and thus ICU beds occupied), as well as patient transfers to higher-level facilities may have affected this relationship.

In the mean ICU occupancy model, interestingly, we found that the proportion of residents aged 65+ is a significant predictor in the multiple regression model across all time periods (negative coefficient estimates). This finding should be interpreted with caution, as there could be one or more confounding factors that may have affected this relationship. Namely, the proportion of ICU beds occupied is not simply a function of demand, but rather supply and demand, as some counties have different ICU capacities. Further, these findings may be influenced by several outlier counties within Florida and Texas (and Fresno, California). Also, the mean ICU occupancy model accounts for all ICU admissions and not just COVID-19. These factors indicate that the percentage of population aged 65+ does not tell the whole story when predicting ICU occupancy and it is important to consider factors beyond this demographic variable when estimating the ICU occupancy of a county.

Overall, in the case of severe outcomes, on a macro-level, while communities with higher median household income tended to have less deaths during Delta 2 and Omicron, population density was shown to have differing effects in these respective time periods. In Delta 2, a higher population density was negatively associated with deaths, possibly because of advanced healthcare infrastructure in densely populated areas. However, the positive relationship between population density and deaths in Omicron again corroborates the CDC report that a surge in cases (from Omicron’s highly transmissive nature) may lead to significant increases in deaths (2). Age may also play an important role in the macro-level outcomes of a community, as the proportion of residents aged 65 and older were associated with deaths. Altogether, these findings suggest that communities with relatively less median household income and communities with a higher older adult population may be affected more severely by COVID-19. It also highlights the change in relationships over the course of different dominant variants, as evidenced by population density’s changing relationship with deaths. These implications may help communities become more aware of possible macro-level outcomes associated with their characteristics and plan accordingly.

Among factors known to affect public health outcomes, the four independent variables chosen in this study have been shown previously to highly influence health outcomes. That is, (1) COVID-19 vaccinations have been shown to be the most effective tool at curtailing spread and severity, (2) communities with lower socioeconomic status tend to fare worse than those with higher socioeconomic status (17–19), (3) communities with high population density may find it harder to social distance, but may also even fare better due to higher quality healthcare systems (24), and (4) individuals aged 65+ are at a higher risk of severe COVID-19 outcomes (23).

We explored this space in our present study and have found significant county-characteristic predictors for both severe and non-severe health outcomes on a macro level in the 50 highest populated counties in the U.S., that may be useful to elucidate the knowledge underlying the trends of COVID-19 and its variants. Namely, we showed that socioeconomic status and the proportion of a population vaccinated may be a large factor in the spread and severity in highly populated U.S. counties.

### 4.2 Limitations of this study

#### 4.2.1 Overall data

Reporting standards across different sources may slightly differ. However, we did the best we could to ensure the validity of the publicly available data.

#### 4.2.2 Missing data points

King County, Washington—Positivity rate in Delta 1 and 2, and Omicron. Riverside County, California—Infection rate in Delta 1 and 2, and Omicron. Montgomery County, Maryland—Infection rate in Delta 1 and 2. Collin County, Texas—Infection rate in Delta 1 and 2. Florida counties—Deaths in Delta 1.

#### 4.2.3 Sample

The dynamics of the spread and severity may have been different in less populous counties that were not included in this study.

#### 4.2.4 Other

There may have been other factors affecting non-severe and severe health outcomes like travel patterns and granular case-by-case health policies toward COVID-19. During the span of the data collected (20 June 2021 to 19 March 2022), much of the previously enacted lockdowns in the U.S. were lifted or were not as stringent or widespread as from 2020 to early 2021. They were therefore considered to not have been prevalent enough to impact findings. However, other implied stringencies like mask-wearing and social distancing could not be accounted for in our data sources and may have affected relationships depending on compliance and enforcement of such stringencies.

### 4.3 Conclusion

As SARS-CoV-2 continues to evolve, it is vital to understand how the virus’s mutations ultimately affect the health outcomes of populations as well as factors that may influence the rate of transmission or level of severity in a community. Our study was conducted to bring light to the associations of how public health outcomes change over the course of Delta and Omicron’s dominance in the 50 most populous U.S. counties, and how these outcomes differ based on county characteristics. It is important to note that while this study may suggest relationships between certain variables, the underlying parameters affecting the health outcomes of COVID-19 are part of a vastly complex network. Our study aims to lower the obscurity surrounding this complex network in order to more precisely understand the dynamics of COVID-19 spread and severity on a macro-level.

## Data availability statement

The original contributions presented in the study are included in the article/supplementary material, further inquiries can be directed to the corresponding author.

## Ethics statement

Ethical approval was not acquired nor applicable for this research article. No human or animal participants were involved in the study. Publicly available data was used in the process of this study.

## Author contributions

AB: conceptualization, methodology, software, formal analysis, investigation, data curation, writing—original draft, and writing—review and editing. YZ: conceptualization, investigation, writing – original draft, and writing—review and editing. SS: conceptualization, writing—original draft, and writing—review and editing. AA: conceptualization, writing—original draft, and writing—review and editing. DD: conceptualization, writing—original draft, writing—review and editing, supervision, project administration, and funding acquisition. All authors contributed to the article and approved the submitted version.
