# Supplementary material for: Relationships between COVID-19 healthcare outcomes and county characteristics in the U.S. for Delta (B.1.617.2) and Omicron (B.1.1.529 and BA.1.1) variants
Source: Front Public Health. 2023 Nov 17;11:1252668. doi: 10.3389/fpubh.2023.1252668 (PMC10693294; doi:10.3389/fpubh.2023.1252668)
Supplement: Supplementary file 1 [file Table_1.DOCX]

Table A.1. Correlation coefficients (R) and p-values (p) of each dependent and independent variable combination in each time frame.

Note: Vax = mean proportion of residents with 2+ dose, MHI = median household income, PD = population density, 65+ = Proportion of residents aged 65 and older

|  | **Vax R** | **Vax - p** | **MHI - R** | **MHI - p** | **PD - R** | **PD - p** | **65+ - R** | **65+ - p** | **n** |
| --- | --- | --- | --- | --- | --- | --- | --- | --- | --- |
| **Delta 1** |  |  |  |  |  |  |  |  |  |
| meanCaseDensityDelta1 | -0.290 | 0.044 | -0.390 | 0.005 | -0.230 | 0.110 | 0.073 | 0.610 | 50 |
| meanNewDeathsPer100kDelta1 | -0.520 | 0.000 | -0.480 | 0.001 | -0.220 | 0.170 | -0.320 | 0.037 | 43 |
| meanPositivityRateDelta1 | -0.490 | 0.000 | -0.430 | 0.002 | -0.350 | 0.013 | -0.039 | 0.790 | 49 |
| meanInfectionRateDelta1 | -0.033 | 0.820 | 0.170 | 0.260 | 0.008 | 0.960 | -0.085 | 0.570 | 47 |
| meanICUOccupanyDelta1 | -0.350 | 0.014 | -0.280 | 0.048 | -0.130 | 0.370 | -0.360 | 0.011 | 50 |
| meanHospitalizationsPer100kDelta1 | -0.390 | 0.005 | -0.430 | 0.002 | -0.190 | 0.180 | 0.028 | 0.850 | 50 |
| meanPropIcuFromCovidDelta1 | -0.260 | 0.065 | -0.270 | 0.056 | -0.370 | 0.008 | -0.096 | 0.510 | 50 |
|  |  |  |  |  |  |  |  |  |  |
| **Delta 2** |  |  |  |  |  |  |  |  |  |
| meanCaseDensityDelta2 | -0.240 | 0.099 | -0.230 | 0.110 | -0.091 | 0.530 | 0.140 | 0.320 | 50 |
| meanNewDeathsPer100kDelta2 | -0.560 | 0.000 | -0.690 | 0.000 | -0.340 | 0.016 | 0.210 | 0.140 | 50 |
| meanPositivityRateDelta2 | -0.480 | 0.000 | -0.380 | 0.007 | -0.330 | 0.019 | 0.014 | 0.920 | 49 |
| meanInfectionRateDelta2 | 0.310 | 0.031 | 0.200 | 0.180 | 0.500 | 0.000 | 0.210 | 0.160 | 47 |
| meanICUOccupanyDelta2 | -0.340 | 0.017 | -0.220 | 0.120 | -0.007 | 0.960 | -0.480 | 0.000 | 50 |
| meanHospitalizationsPer100kDelta2 | -0.550 | 0.000 | -0.520 | 0.000 | -0.190 | 0.190 | 0.005 | 0.720 | 50 |
| meanPropIcuFromCovidDelta2 | -0.320 | 0.021 | -0.210 | 0.140 | -0.450 | 0.001 | -0.150 | 0.280 | 50 |
|  |  |  |  |  |  |  |  |  |  |
| **Omicron** |  |  |  |  |  |  |  |  |  |
| meanCaseDensityOmicron | 0.410 | 0.003 | -0.076 | 0.600 | 0.410 | 0.003 | 0.052 | 0.720 | 50 |
| meanNewDeathsPer100kOmicron | -0.320 | 0.022 | -0.590 | 0.000 | 0.260 | 0.068 | 0.420 | 0.002 | 50 |
| meanPositivityRateOmicron | -0.650 | 0.000 | -0.460 | 0.001 | -0.370 | 0.008 | -0.170 | 0.240 | 49 |
| meanInfectionRateOmicron | -0.005 | 0.970 | 0.210 | 0.150 | -0.300 | 0.040 | -0.130 | 0.370 | 48 |
| meanICUOccupanyOmicron | -0.350 | 0.012 | -0.190 | 0.180 | -0.032 | 0.820 | -0.570 | 0.000 | 50 |
| meanHospitalizationsPer100kOmicron | -0.300 | 0.034 | -0.430 | 0.002 | 0.500 | 0.000 | 0.073 | 0.610 | 50 |
| meanPropIcuFromCovidOmicron | -0.150 | 0.280 | -0.082 | 0.570 | -0.004 | 0.980 | -0.240 | 0.092 | 50 |
